# The Effect of In Vitro Digestion on Antioxidant, ACE-Inhibitory and Antimicrobial Potentials of Traditional Serbian White-Brined Cheeses

**DOI:** 10.3390/foods8030094

**Published:** 2019-03-12

**Authors:** Miroljub Barac, Tanja Vucic, Sladjana Zilic, Mirjana Pesic, Marina Sokovic, Jovana Petrovic, Aleksandar Kostic, Ivana Sredovic Ignjatovic, Danijel Milincic

**Affiliations:** 1 Faculty of Agriculture, University of Belgrade, Nemanjina 6, 11081 Belgrade, Serbia; tvucic@agrif.bg.ac.rs (T.V.); mpesic@agrif.bg.ac.rs (M.P.); akostic@agrif.bg.ac.rs (A.K.); isredovic@agrif.bg.ac.rs (I.S.I.); danijel.milincic@agrif.bg.ac.rs (D.M.); 2 Maize Research Institute, Slobodana Bajica 1, 11081 Belgrade, Serbia; sladjana.zilic505@gmail.com; 3 Institute for Biological Research “Sinisa Stankovic”, University of Belgrade, 11000 Belgrade, Serbia; mris@ibiss.bg.ac.rs (M.S.); jovana0303@ibiss.bg.ac.rs (J.P.)

**Keywords:** white-brined cheeses, in vitro digestion, functionality

## Abstract

This study deals with the effect of in vitro digestion on the functional potential of traditional Serbian white-brined cheeses. The total antioxidant capacity, reducing power and iron (II) chelating properties as well as angiotensin-converting enyzme-inhibitory (ACE-inhibitory) and antimicrobial activities of traditional Serbian white-brined cheeses before and after in vitro digestion were assayed. The traditional cheeses had different antioxidant properties as well as different ACE-inhibitory activities. In vitro digestion improved the total antioxidant capacity (8.42–58.56 times) and the reducing power (by 17.90–99.30%) of investigated cheeses, whereas their chelating ability was slightly improved or unaffected after digestion. In vitro digestion reduced the ACE-inhibitory potential of water-soluble protein fractions, and digested water-insoluble fractions were the major source of ACE-inhibitory peptides. The digestates did not exhibit any antibacterial potential, whereas they showed moderate antifungal potential toward selected micromycetes. The best antifungal potential had Svrljig ovine cheese and Homolje cow cheese. The results of this study clearly point to a significant functionality of traditional white-brined cheeses.

## 1. Introduction

Based on the “working definition” of FUFOSE (Functional Food Science in Europe), a food can be regarded as “functional” if it is satisfactorily demonstrated to beneficially affect one or more target functions in the body beyond adequate nutritional effects in a way that is relevant to either an improved state of health and well-being and/or a reduction of risk of disease [1]. Functional foods must remain foods, and they must achieve their effects in amounts normally consumed in a diet [2].

Traditional white Serbian cheese, usually named as “Krishka cheese”, is the most widely manufactured and consumed cheese variety in Serbia. Given that it represents about 60% of the total cheese production in Serbia, this cheese has a considerable input in the economy, being significant in the income of milk producers, especially those in rural areas who do not have access to milk processing plants. Additionally, it also has a significant influence on local human nutrition. Traditional “Krishka cheese” is artisanally produced from raw cow or sheep milk without the addition of a starter culture and is named according to the region of production (Homolje, Zlatar, Svrljig and Sjenica cheese). The specificity of “Krishka cheese” is that ripening takes place in brine usually within 1–3 months. Each cheese maker has an individualized method for all steps in the production, especially regarding pressing and salting. Therefore, the chemical characteristics of cheeses produced in the same region from the same type of milk can vary significantly. However, the main features of this cheese are its high acidity and sharp and salty flavour.

Studies conducted over the past fifteen years have shown that cheeses are potentially good sources of bioactive proteins and peptides. Part of these proteins and peptides originates from milk itself, but most of them are released during cheese production [3,4,5,6] and digestion [7]. Today, it is known that the level of their formation during cheese processing is determined by numerous factors including the heat treatment conditions of milk and the cheese ripening conditions (type and activity of proteolytic agents, conditions and duration of ripening process) [8].

Considering the favourable healthy indices related to fatty acids composition and mineral profiles of traditional “Krishka cheese” [9], the aim of the present study was to investigate its bioactivity as an antioxidant and its angiotensin-converting enyzme-inhibitory (ACE-inhibitory) and antimicrobial activities in order to examine its potential as a functional food.

## 2. Materials and Methods 

### 2.1. Cheese Samples

This study covers four Serbian traditional white-brined cheeses (Zlatar, Sjenica, Svrljig and Homolje cheeses) prepared from sheep and cow milk. Ripened (two-month-old) artisanal cheeses were collected (at least three different samples of each type of cheese from the same producer) from a specific area during the period from April to September. The collected samples were transported to the laboratory in an isothermal container and kept at −20 °C until analysis. 

### 2.2. Chemical Characteristics of Cheeses

Prior to the chemical analysis, the cheese samples (whole slices) were ground to achieve uniformity. The chemical composition of the cheese samples was determined using the following methods: dry matter by the standard drying method at 102 ± 2 °C [10]; fat content according to the method of Van-Gulik [11]; nitrogen content by the Kjeldahl method [12]; protein content was calculated as the nitrogen content multiplied by 6.38; and NaCl content by the Volhard method [13]. Based on these characteristics, the following parameters were calculated: fat in dry matter (FDM) and moisture in nonfat solids (MNFS). The pH of grated cheese slurried with an equal volume of water was measured using a pH meter (Consort, Belgium) [14]. The water-soluble nitrogen content (WSN) was prepared according to the method of Kuchroo and Fox [15]. Nitrogen soluble in 12% trichloracetic acid, TCA (TCA-SN: content of nitrogen soluble in trichloracetic acid) and in 5% phosphotungistic acid, PTA (PTA-SN: content of nitrogen soluble in phosphotungistic acid) was prepared from the water-soluble fraction. The ripening index (RI) was expressed as WSN/TN (content of water-soluble nitrogen/total nitrogen content) × 100 as suggested by Kuchroo and Fox [15]. All of these parameters were determined by the Kjeldahl method [12]. All determinations were made in triplicate.

### 2.3. Preparation of Water-Soluble and Water-Insoluble Fractions

The water-soluble (WSF) and water-insoluble protein fractions (WINF) of cheese were separated according to the following procedure: a grounded cheese (15 g) was extracted in 45 mL of Ultrapure water (Ultrapure water system, SG ver.1.11, Waters, Milford, MA, USA) tempered at 40 °C. To preserve the extract, a drop of formaldehyde was added. The extraction was carried out in an ultrasound bath (Clifton, UK) for 90 min. After that period, the extract was cooled in the freezer for 1 h and centrifuged for 15 min at 4000× *g* (Janetzki, Prague, Czech Republic). Then, the upper layer was carefully removed, and the supernatant was filtered through Whatman No 1. To further remove any impurities, the obtained filtrate (WSF) was filtered through a 0.45-µm-pore-size filter (Millipore, Billerica, MA, USA) and lyophilized. The precipitate (WINF) was rinsed out with three portions of 5 mL of Ultrapure water. To remove any residual lipids, the WINF was treated with n-hexane for one hour, filtered through Whatman No.1, dried at room temperature overnight and lyophilized. 

### 2.4. In Vitro Simulated Digestion 

Whole cheeses and their protein fractions were subjected to in vitro gastrointestinal digestion as described by Petrat-Melin et al. [16]. Briefly, the digestion consisted of a two-step static system with a simulated gastric phase using porcine pepsin (Sigma Aldrich, St. Louis, MO, USA) at pH 2.0 for 60 min, followed by a simulated duodenal phase. In the duodenal phase, the pH was increased to 6.5 by the addition of 55 mM of NaHCO_3_, and digestion was carried out for 120 min with porcine pancreatin (Sigma Aldrich). Equal enzyme activities were used for both steps, corresponding to a *w*/*w* ratio of enzyme to protein of approximately 1 to 200. After digestion, the enzymes were inactivated by a heat treatment at 90 °C for 5 min and immediately cooled in an ice bath. Before digestion and after each step of digestion, 50 µL of the reaction mixture was sampled and diluted with a sample buffer (Tris-HCl pH 6.6) for sodium dodecil sulfate-electrophoresis (SDS-electrophoresis) and then frozen at −20 °C. 

### 2.5. Sodium Dodecil Sulfate Polyacrilamide Gel Elecrophoresis (SDS-PAGE)

The digestion process was followed by an SDS-electrophoresis according to the method of Fling and Gregerson [17] as previously reported by Barac et al. [5,6]. An SDS-PAGE was performed on 12.5% resolving gels and 5% stacking gels. The gels were run at 30 mA per gel for 4 h to completion and stained with 0.23% (wt/vol) Coomassie Blue R-250 (dissolved in 3.9% trichloroacetic acid (TCA), 6% acetic acid and 17% methanol) for 45 min. Then, the gels were destained with 8% acetic acid and 18% ethanol. The molecular weight of the polypeptides was estimated by using the low molecular weight calibration kit (Pharmacia, Upsalla, Sweden). The molecular weight marker included the following: phosphorylase B (94.0 kDa), bovine serum albumin (67.0 kDa), ovalbumin (43.0 kDa), carbonic anhydrase (30.0 kDa), soybean trypsin inhibitor (20.1 kDa) and a-lactalbumin (14.4 kDa). 

### 2.6. Total Free Amino Acid Level 

The total free amino acid levels of whole cheeses before and after in vitro digestion were determined by the method of Hayaloglu [18]. Lyophilized cheese samples (0.5 g) were extracted in 10 mL of MilliQ water and filtered through a 0.45-µm-pore-size filter (Millipore, Billerica, MA, USA). The same quantity of lyophilized digestates (0.5 g) was dissolved in 10 mL of MilliQ water and filtered. A quantity of 100 μL of the filtrate was diluted into 1 mL with H_2_O, and 2 mL of a Cd-ninhydrin reagent (0.8 g ninhydrin was dissolved in a mixture of 80 mL ethanol and 10 mL glacial acetic acid, followed by the addition of 1 g CdCl_2_ dissolved in 1 mL of distilled water) was added. The Mixtures were vortexed and heated at 84 °C for 5 min and cooled in ice-water, and the absorbance was determined at 507 nm. The results were expressed as miligram Leu per gram of lypohilized WSF by reference to a standard curve which was first prepared using Leu (Sigma Chemical Co., St Louis, MO, USA) at various concentrations (0.050–0.500 mg Leu mL^−1^ water).

### 2.7. Total Antioxidant Capacity

The total antioxidant capacity (TEAC) of undigested and digested cheeses was measured according to the QUENCHER method (QUick, Easy, New, CHEap and Reproducible method) [19] which is based on the direct measurement of solid samples by mixing them with the free radicals followed by a subsequent spectrometric measurement. As the stock solution, 7 mM of an aqueous solution of ABTS (2,2-azino-bis/3-ethil-benothiazoline-6-sulfonic acid) with 2.45 mM K_2_O_8_S_2_ was used. The working solution of ABTS•+ was obtained by diluting the stock solution in a water/ethanol (50:50, vol/vol) solution. Ground lyophilized samples (6 mg) were mixed with 20 mL of the ABTS•+ working solution, and the mixture was rigorously shaken for 25 min in a cold room at 4 °C. After centrifugation at 9200× *g* for 5 min at 10 °C, the absorbance measurement was performed at 734 nm. The total antioxidant capacity was calculated by means of a calibration curve built in a range between 0.5 and 5 µg of Trolox per mL of the ABTS•+ solution and expressed as the Trolox equivalent antioxidant capacity (TEAC) in mmol of Trolox per kg of dry matter (DM).

### 2.8. Reducing Power

The reducing power of digested and undigested cheeses was assessed as suggested by Meira et al. [20]. Briefly, lyophilized samples were dissolved in a 0.2 M phosphate buffer with pH 6.6 (15 mg/mL). A volume of 2.5 mL was added to 2.5 mL of 10 mg/mL potassium ferricyanide (Sigma, Aldrich). The mixture was incubated at 50 °C for 20 min. TCA (2.5 mL, 10% *w*/*v*) was added to the mixture, which was then centrifuged at 3000× *g* for 10 min. The supernatant (2.5 mL) was mixed with 2.5 mL distilled water, and after the addition of 0.5 mL of 1 mg/mL ferric chloride (l), the absorbance was measured at 700 nm. A higher absorbance of the reaction mixture indicates a greater reducing power. Butylated hydroxytoluene (BHT, Sigma-Aldrich) at the same concentrations as the samples was used as a positive control.

### 2.9. Iron (II) Chelating Activity Assay

The ferrous ion chelating ability of cheese before and after in vitro digestion was determined using a slightly modified ferrozine method [21]. Digested and non-digested cheeses were dissolved in a 0.2 M phosphate buffer with pH 6.6 at four different concentrations (20, 30, 40, 50 and 75 mg/mL). A volume of 1 mL of each solution was mixed with 3.7 mL of distilled water, 0.1 mL of 2mM FeSO4 and 0.2 mL of 5mM ferrozine (3-(2-pyridyl)-5,6-bis(4-phenyl-sulfonic acid)-1,2,4-triazine, Sigma-Aldrich). After 10 min, the absorbance of the reaction mixture was read at 562 nm. As a control, 1 mL of the 0.2 M phosphate buffer with pH 6.6, instead of the sample, was used. Ethylenediamine tetraacetic acid (EDTA, 50 mg/mL, Sigma-Aldrich) was used as a positive standard. The chelating activity for each concentration was calculated as follows:

Chelating activity (%) = (1 − (Absorbance of sample/Absorbance of control)) × 100.

The chelating activity was plotted against the sample concentration, and the IC_50_ for each protein fraction was determined. IC_50_ represents the concentration (mg/mL) of proteins that is required for 50% of ferrous ions chelating.

### 2.10. ACE-Inhibitor Activity

The ACE-inhibiting potential is associated with low molecular weight peptides. Thus, the ACE-inhibitory activities of the WSF fraction before and after digestion and of WINF after digestion were determined. The ACE-inhibitory activity was measured according to a modified method of Otte et al. [22] by using furanacroloyl-Phe-Glu-Glu (FA-PGG) as a substrate. Briefly, an ACE enzyme solution was prepared from rabbit lung acetone powder (Sigma-Aldrich, St. Louis, MO, USA) as described by Vermeirssen et al. [23]. Digested (WSF and WINF) and undigested (WSF) peptide samples (20 mg) were dissolved in 2 mL of MilliQ water and centrifuged at 16,000× *g* for 5 min, and then, supernatants were filtered through a 0.45-µm-pore-size cellulose acetate filter (Millipore, Billerica, MA, USA). Peptide solutions (10 mg/mL) and four dilutions (0.5 mg/mL, 1 mg/mL, 5 mg/mL and 7.5 mg/mL) were used for the ACE-inhibitory activity. Aliquots of 10 µL of peptide solution and 300 µL of preheated (37 °C, 15 min) substrate solution (0.88 mM Fa-PGG in 50 mMTris-HCl buffer pH 7.5) were mixed with 1 mL of diluted and preheated (37 °C, 15 min) ACE enzyme solution (0.025 U/mL) in cuvette, and the absorbance at 340 nm was measured immediately. The absorbance was recorded every 60 s for 20 min. Three replicates of each diluted sample were made. The control samples contained water instead of the inhibitor solution. The ACE activity was expressed as the slope of the decrease in absorbance at 340 nm (rA), and the ACE inhibition (%) was calculated from the ratio of the slope in the presence of the inhibitor to the slope obtained in the absence of the inhibitor, according to the formula

% ACE inhibition = (1 − (rAinhibitor/rAcontrol)) × 100

The inhibitor concentrations that inhibit ACE by 50% in comparison to the control (IC_50_ values) were determined from plots of % ACE inhibition versus inhibitor concentration.

### 2.11. Antimicrobial Potential of Digestates Antimicrobial Activity 

For the evaluation of the antimicrobial activity of digested protein fractions and cheeses, Gram negative bacteria—*Enterobacter cloacae* (ATCC 35030), *Escherichia coli* (ATCC 35210), *Pseudomonas aeruginosa* (ATCC 27853) and *Salmonella typhimurium* (ATCC 13311)—and Gram positive bacteria—*Staphylococcus aureus* (ATCC 6538), *Bacillus cereus* (clinical isolate), *Micrococcus flavus* (ATCC 10240) and *Listeria monocytogenes* (NCTC 7973)—were used, as well as selected micromycetes: *Aspergillus fumigatus* (ATCC 1022), *Aspergillus ochraceus* (ATCC 12066), *Aspergillus versicolor* (ATCC 11730), *Trichoderma viride* (IAM 5061), *Penicillium ochrochloron* (ATCC 9112) and *Penicillium funiculosum* (ATCC 36839). The microorganisms were obtained from the Mycological Laboratory, Department of Plant Physiology, Institute for Biological Research “Siniša Stanković”, University of Belgrade, Serbia. 

In order to determine the minimum inhibitory (MIC) and minimum bactericidal/fungicidal (MBC/MFC) concentrations, a microdilution method in plates with a flat bottom (96 system) was used according to Soković [24]. Fresh cultures of bacteria (1.0 × 10^9^ cells/mL) were obtained after an overnight cultivation of selected bacteria in Tryptic Soy Broth (Torlak, Belgrade) at 37 °C. An inoculum was adjusted with a sterile saline solution to a concentration of 1.0 × 10^6^ cells/mL in a final volume of 100 μL per well and stored at 4 °C until further use. Dilutions of inocula were cultured on a solid medium to verify the absence of contamination and to ensure the validity of the inoculum. Compounds dissolved in 5% DMSO were added to the nutrient broth, after which bacterial inoculums were added, and plates were incubated for 24 h at 37 °C. As a positive control, the growth of the pathogen without the addition of potentially inhibitory compounds was observed. The lowest concentration of the tested compound that significantly reduced the growth of bacteria in comparison to the positive control (under binocular microscope) was defined as the MIC value. The results were confirmed after adding 40 μL of the purple indicator color *p*-iodonitrotetrazolium chloride I8377-Sigma (0.2 mg/mL of distilled water) to each well and further incubating for 30 min at 37 °C. A red-violet color of the well’s contents indicated the growth of the tested bacteria [25]. The complete absence of growth, i.e., the determination of the MBC value was evaluated after the reinoculation of 10 μL of the well content with no bacterial growth into 100 μL of sterile nutrient broth and its incubation for 24 h at 37 °C. As for the evaluation of antifungal activity, the microdilution method was adjusted as follows: the spore suspension of tested micromycetes was prepared by washing 21-day-old fungal cultures with 0.85% sterile saline containing 0.1% Tween 80 (*v*/*v*). The number of spores was determined with a Neubauer hemocytometer and adjusted with sterile saline to a concentration of approximately 1.0 × 10^5^ cells/mL in a final volume of 100 μL per well. Dilutions of the inocula were cultured on the solid malt agar (MA) to verify the absence of contamination and to check the validity of the inoculum. The MIC values were evaluated by a serial dilution technique in microtiter plates. Selected compounds were added to a broth malt medium, after which fungal inocula were added. The plates were incubated for five days at 25 °C. The lowest concentrations with a reduced mycelial growth in comparison to the positive control were defined as the MIC values (under binocular microscope), while the MFC values were determined after the reinoculation of the well content where no fungal growth was observed onto the sterile agar plates and their further incubation for 72 h at 25 °C. 

### 2.12. Statistical Analysis

Due to different characteristics induced mainly by individualized cheese making processes and types of milk analyzed, cheeses were observed as a specific variety of white-brined cheese. All measurements were performed in triplicate. The data were subjected to a two-way analysis of variance (ANOVA) with IBM-SPSS v20 software (IBM Corp., Armonk, NY, USA), and the comparison of means was done by Tukey’s test at *p* < 0.05. A Principal Component Analysis (PCA) was performed using the Pearson correlation as suggested by Barac et al. [5,6]. 

## 3. Results and Discussion

### 3.1. Chemical Characteristics of Cheeses

Traditional Serbian white cheeses in brine are produced from raw milk without the addition of a starter culture and are named according to the region of production (Homolje, Zlatar, Svrljig and Sjenica cheese). Each cheese maker has an individualized method for all steps in the production, especially regarding pressing and salting. Therefore, the chemical characteristics of cheeses produced in the same region from the same type of milk can vary significantly. Table 1 shows the gross composition of traditional Serbian white-brined cheeses. According to the fat in dry matter content (FDM) and the Codex standard 208–1999 [26], Sjenica ovine cheese belonged to the group of high fat cheeses, whereas all other cheeses belonged to full fat cheeses. Significant differences (*p* ˂ 0.05) in FDM were observed between cheeses, but all values were in the range reported for Turkish white-brined cheeses [27]. Regarding the moisture in nonfat solids (MNFS), Homolje cheeses prepared from cow and ovine milk belonged to the group semihard cheeses, while other cheeses were classified as soft cheeses. Cow cheeses from Sjenica and Zlatar had higher MNFS comparing to Turkish white-brined cheeses [28,29]. The ripening index (RI) of the investigated cheeses varied. The highest RI was found in Zlatar cheese which may be due to lower NaCl and DM contents. Generally, the significant differences (*p* ˂ 0.05) which were observed among different cheeses could be attributed to numerous factors such as milk type; composition of the raw milk depending on race, feed and the production system; autochthonous microflora; and diversity in the technology used in their manufacturing. 

### 3.2. Antioxidant Properties of Cheeses and Their Digestates

The antioxidative properties of cheeses and their digestates were evaluated by three different methods: total radical scavenging activity, reducing power and iron (II) chelating ability. These results are presented in Table 2. 

The total radical scavenging capacities of cheeses (expressed as TEAC) before digestion were different. Also, significant differences (*p* ˂ 0.05) between the TEAC values of cheeses were observed after their in vitro digestion. Homolje cheese prepared from sheep and cow milk and Zlatar cheese prepared from cow milk demonstrated the smallest values of TEAC, whereas Svrljig cheese before digestion was the most efficient for radical scavenging. The average antioxidant capacities of these cheeses were 5.82, 8.81, 10.28 and 36.43 mmol Trolox Eq/kg (Table 2).

Due to methodological differences and insufficient data about the scavenging capacity of whole cheeses, inter-study comparisons with other types of cheeses are hard to conduct. Namely, most of the studies conducted in this field were related only to the antioxidant properties of the water extract of cheeses. In our previous studies [5,6], we showed that the antioxidant capacity of insoluble fractions which represent the majority of cheese proteins (about 80% of total proteins) could not be ignored. However, some of the investigated cheeses such as Svrljig sheep milk cheese and cow and sheep milk cheeses from Sjenica had TEAC values which were similar to the values of plant-based protein isolates reported by Žilić et al. [30] and Di Benedeto et al. [31].

Cheese is a complex food system composed of a protein matrix in which different components including fat, fatty acids, peptides, free amino acids, vitamins, minerals and polyphenolic compounds are chemically, non-covalently or physically incorporated. In such systems, the radical scavenging activity cannot be considered as the simple summation of the TEACs of their constituents [32]. According to Cömert and Gökmen [33], the coexistence of multiple antioxidants usually results in additive, synergistic or antagonistic interactions.

Major contributors to radical scavenging capacity of cheeses are proteins (caseins and residual whey proteins), peptides and free amino acids released during ripening [34,35,36,37]. The antioxidant capacity of proteins and peptides depends on numerous factors including the amino acid position in the protein sequence, their physical structure, hydrophobicity and their molecular weight [37,38]. Medina-Navarro et al. [39] emphasized that the structural molecular integrity is the most important issue for proteins if they work as antioxidants. Although the effect of phenols, fat-soluble antioxidants and other minor constituents in milk was found to be negligible [40], their impact on the TEAC of cheese cannot be ignored [41,42].

**Figure 1:**
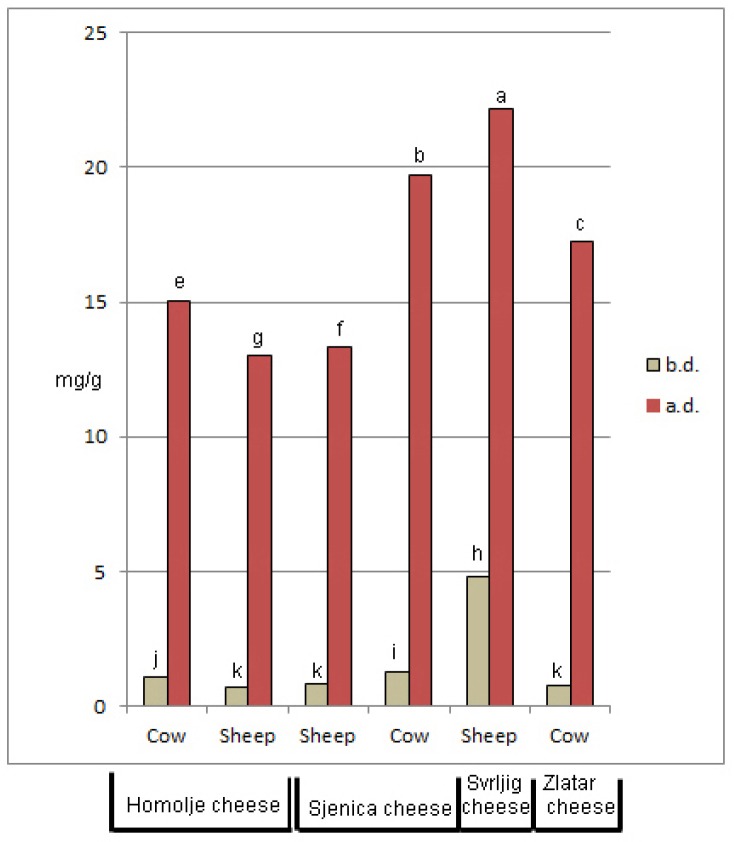
The free amino acid contents of the cheeses before and after in vitro digestion. Data marked with different lowercase letters are significantly different at *p* ˂ 0.05; b.d., before digestion; a.d., after digestion.

The investigated cheeses had significantly (*p* ˂ 0.05) different TP (total protein)/DM, TCA-SN and PTA-SN content (Table 1) and free amino acid content (Figure 1). Furthermore, it was shown that these cheeses had significantly different contents and compositions of fat as well as different mineral contents [9]. Therefore, the differences observed between TEACs could be mainly attributed to the abovementioned factors and their interactions.

**Figure 2:**
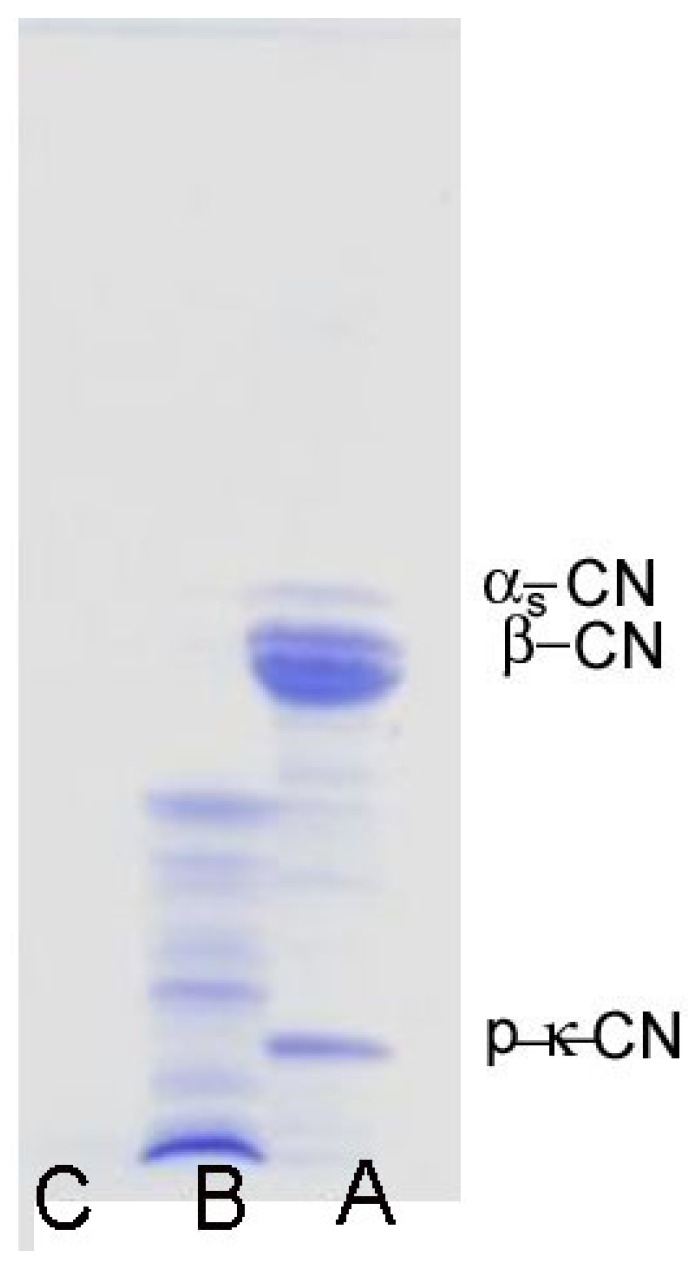
Sodium dodecyl sulfate–polyacrylamide gel electrophoresis (SDS-PAGE) of whole Sjenica sheep cheese before digestion (**A**), after pepsin digestion (**B**) and after pepsin + pancreatin digestion (**C**).

In vitro digestion significantly (*p* ˂ 0.05) improves the TEAC of all investigated cheeses. The average TEAC values were in the range of 250.4–400.1 mmol Trolox Eq/kg (Table 2). In other words, in vitro digestion improved the initial TEACs of cheeses by 8.42–58.56 times. The most intensive increase was observed in the case of Homolje sheep milk cheese, and in general, the highest radical scavenging capacities had all investigated sheep milk cheeses. For example, the average TEACs of sheep milk cheeses (306.94–400.1 mmol Trolox Eq/kg) were approximately two times higher than the TEAC of Sjenica cow milk cheese. Such an increase of TEAC after simulated in vitro digestion could be attributed to the intensive hydrolysis of cheese proteins, to the liberation of free amino acids and to low molecular weight peptides that cannot be detected by SDS-PAGE (Figure 2). This is supported by the results related to the change in free amino acid content caused by in vitro digestion (Figure 1). Digestion dramatically increased the free amino acid contents up to 20 times approximately. Strong radical scavenging of low molecular weight peptides from different cheeses is well known [20,43]. Also, it is known that almost all amino acids can interact with highly energetic free radicals [30,44]. In addition, the improved antioxidant capacity could be also attributed to liberated indigenous phenols [45], fat-soluble antioxidants and other minor compounds which were incorporated in the cheese protein matrix [46].

The majority of the reducing power of cheeses ranged from 0.1 to 0.2 units of absorbance at 700 nm (Table 2). Values out of this range were observed only for cow cheese from Sjenica. In general, undigested cow milk cheeses had a slightly higher reducing power (0.156–0.296) than sheep milk cheeses (0.136–0.150). In vitro digestion improves the reducing power of cheeses for 17.90–99.30%. After digestion, the reducing power of almost all cheeses were in the range of 0.2–0.3 units, suggesting that they are the source of protons and electrons to maintain such a redox potential at the investigated concentration. Values out of this range were observed for cow milk cheese from Homolje (0.194) and from Sjenica (0.349). BHT exhibited a much higher absorbance of 1.624 at the same concentration as for analyzed cheeses.

The differences observed between the reducing power of undigested cheeses could be attributed to the different content of two types of cheese compounds, those that originated from milk and those that formed as a result of microbial acting [46]. Several compounds in cheese that originate from milk are known to have a high ferric-reducing ability, e.g., α-tocopherol, ascorbic acid, whey proteins, caseins and minerals [47]. In addition, fatty acids that are not antioxidant per se may be indirectly associated with the reducing power of some cheese varieties due to a primary correlation with other antioxidant compounds (e.g., vitamin E and β-carotene) [46]. Also, compounds formed as a result of microbial activity, such as folate, known to be antioxidant and able to reduce Fe^3+^ to Fe^2+^ could contribute to reducing power of cheese [46,47]. Besides proteolysis, the liberation of these compounds during in vitro digestion could be one of the main reasons for the improved reducing power. 

Traditional white cheeses in brine had different iron (II) chelating abilities. Depending on the cheese variety, the before digestion values of IC_50_ ranged from 15.19–42.34 mg/mL. The cow cheese from Sjenica showed the best performance. The IC_50_-values for these cheeses were 15.19 mg/mL and 20.63 mg/mL, respectively (Table 2). The other cheeses demonstrated a much lower ability for iron binding. The iron chelating ability of cheeses depends on several factors. It is known that the iron chelating ability is related to the presence of aromatic and hydrophobic amino acids, especially great amounts of histidine (due to the presence of an imidazole ring), phosphoserine residues (contain a polar and anionic domain that is favourable for sequestering cationic metal) and carboxylate groups [48]. In addition, in our previous work [9], we showed that these cheeses contained different initial amounts of metal ions that can also have an influence on the ability to add the chelating properties of cheese.

In vitro digestion affected the iron(II) chelating ability of cheeses differently. Digestion had no significant influence (at *p* ˂ 0.5) on the chelating ability of sheep milk cheese from Sjenica and Svrljig, whereas the chelating ability of other cheeses was lower than the undigested counterpart. It seems that intensive proteolysis reduced the chelating ability of cheeses, which is in a good agreement with the results of Corrêa et al. [49] and Meira et al. [20]. Both groups of authors suggested that the chelating activity of cheese proteins decreased as the hydrolysis degree increased. It has been reported [50] that the major peptides formed during in vitro and in vivo digestion which act as mineral solubilizers and/or carriers are caseinophosphopeptides. These peptides contain high polar acidic sequences of three phosphoserines followed by two glutamic acid residues, which are the binding sites for minerals [51]. However, a closer characterization of the peptides responsible for iron chelating ability after digestion is essential.

Regardless of the observed differences, cheese digestates showed an effective capacity for Fe (II) chelation. This is of great importance because, besides having other properties, the “Krishka cheese” can promote iron absorption and can improve iron bioavailability.

### 3.3. Principal Component Analysis

**Figure 3:**
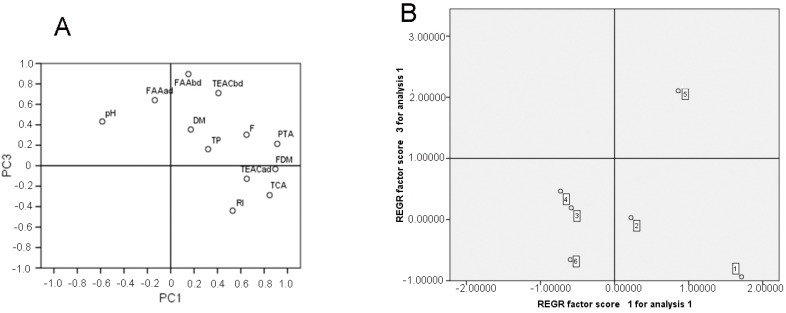
The principal component analysis (PCA) of Serbian traditional white cheeses: PC1 × PC3 of the parameters (**A**) and samples 1–6 (**B**) are given in Table 1 and Table 2. The samples: 1. Sjenica sheep cheese; 2. Sjenica cow cheese; 3. Homolje sheep cheese; 4. Homolje cow cheese; 5. Svrlig sheep cheese; and 6. Zlatar cow cheese. TP, total protein content; DM, dry matter; RI, ripening index; TEAC, Trolox equivalent antioxidant capacity. FAA, free amino acids; PTA, content of nitrogen soluble in phosphotungistic acid; TCA, content of nitrogen soluble in trichloracetic acid. REGR-regression.

The principal component analysis (PCA) performed with the data gathered from the physicochemical analysis of cheese samples and the TEAC values are shown in Figure 3. Three main components (PC1, PC2 and PC3) accounted for 81.69% of the data with a range of 34.76%, 25.62% and 21.31% for the first, second and the third components, respectively. PC1 was associated positively with TCA, PTA and RI of ripening which reflected the degree of proteolysis of the cheese. Also, it was positively associated with TEAC after digestion and FDM. PC2 highlighted a positive association between fat content and TP, whereas PC3 was positively associated with TEAC before digestion, free amino acids (FAA) before digestion and FAA after digestion. Based on the close grouping, it was evident that PTA and TEAC after digestion showed an association with each other. Therefore, it could be predicted that these factors will positively influence the antioxidant capacity of digested cheese by exerting a positive influence on the depended component PC1. Through PC1 and PC3, it is possible to separate the samples into three groups. Samples 1 and 2 (ovine and cow cheese from Sjenica) are allocated on the positive quadrant of PC1, identified by most of the characteristics related to the parameters of proteolysis. The negative quadrant of PC1 generated a second group consisting of samples 3, 4, and 6 highly influenced by low values of TCA, PTA and RI. PC3 generated the third group which consisted only of sample 5 (ovine cheese from Svrljig) influenced by a high content of free amino acid content before and after digestion as well as with a high TEAC value before digestion. 

### 3.4. ACE-Inhibiting Potential of Protein Fractions of Traditional Serbian Cheeses

The ACE-inhibiting potential is associated with low molecular weight peptides. Thus, the ACE-inhibitory activity of WSF before and after in vitro digestion as well as the inhibitory activity of digested WINF were determined. The obtained results are shown in Table 3. 

The WSFs of traditional cheeses had different ACE-inhibitory activities. Depending on the variety, the IC_50_ of undigested WSFs ranged from 2.26 to 4.61 mg/mL. In general, cow cheeses had a better ACE-inhibitory potential than those prepared from sheep milk. The best ACE-inhibitory activity had a WSF fraction of Zlatar cow milk cheese, whereas the lowest ACE-inhibiting potential had a WSF fraction of Sjenica sheep milk cheese. The IC_50_ values were in the range or much lower than those reported by Bütikofer et al. [52] and Sieber et al. [53] for different artisanal cheese varieties. For example, the observed IC_50_ values of WSF of sheep milk cheeses were almost three times lower than those reported by Sieber et al. [53] for WSF of Feta cheese (14.3 ± 1.2 mg/mL). It is known that milk proteins from different species (cow, goat and sheep) during cheesemaking generate almost similar ACE-inhibitory peptides according to their amino acid sequences. However, the results of Gomez-Ruiz et al. [54] and Quereshi et al. [55] indicate that large variations exist in the ACE-inhibitory potential of different cheese varieties as well as within the same type of cheese. This can be attributed to different manufacturing and ripening conditions especially in the absence of a starter-culture and to the activity of specific nonstarter autochthonous microflora [53]. 

In vitro digestion significantly increased the IC_50_ of WSF of all cheeses except Sjenica sheep milk cheese. In other words, due to the extensive proteolytic degradation of peptides responsible for ACE-inhibitory activity, in vitro digestion reduces (almost twice) the inhibitory potential of WSFs. A similar effect of digestive enzymes on the ACE-inhibitory activity of WSF of Emmental cheese was reported by Parrot et al. [54]. Conversely, the average IC_50_ WSF value of Sjenica sheep cheese digestate was almost unchanged, indicating the highest resistance to proteolysis.

The major sources of ACE-inhibitory peptides are caseins which represent the largest part of WINF [55]. Thus, as could be expected, in vitro digestion produced large amounts of ACE-inhibitory peptides. Therefore, as evident from Table 3, after in vitro digestion, WINF had a higher ACE-inhibitory potential than WSF. 

The digested WINF of cow cheeses had a higher ACE-inhibitory potential than the digested WINF of sheep cheeses. The IC_50_ of the digested WINF of cow cheeses was 1.31–4.20 mg/mL, whereas the IC_50_ of the digested WINF of ovine cheeses was 3.28–6.29 mg/mL. The highest inhibitory potential had a WINF of Zlatar cow cheese, and the lowest potential had WINF of Svrljig sheep cheese. The observed differences could be attributed to the different natures of ACE-inhibitory peptides and especially to their different resistances to digestion [56]. 

Evidently, both digested fractions had an ACE-inhibitory potential. However, it should be noted that a high ACE-inhibitory effect in vitro is no guarantee for a hypotensive effect in vivo [57,58]. Peptides have to be bioavailable, to survive digestion and to be transported intact from the intestine to blood, and while there, they must be able to interact and inhibit ACE efficiently. Thus, the further characterization of inhibitory peptide structures and in vivo stability need to be conducted.

### 3.5. Antimicrobial Potential of Cheeses 

The digestates of cheeses and their purified fractions were used to determined the antifungal and antimicrobial (data not shown) activity. The obtained results are presented in Table 4. 

The tested digestates did not exhibit any antibacterial potential, whereas only the presented samples showed a moderate antifungal potential toward selected micromycetes. The best antifungal activity was observed for WINF and whole Svrljig cheese (MIC 2.00 mg/mL, MFC 4.00 mg/mL) against *Aspergillus versicolor* as well as WINF of Homolje cow cheese (MIC 2.08 mg/mL, MFC 4.16 mg/mL) toward the same pathogen. This result may be of importance since this mould is a major producer of hepatotoxic and carcinogenic mycotoxin sterigmatocystin. The WSF of Homolje cow cheese was the only one among the tested that showed a complete inhibition of *Penicillium ochrochloron* and *P. funiculosum* with an MIC value of 2.32 mg/mL and an MFC value of 4.64 mg/mL. *P. funiculosum* produces indol-diterpenoid mycotoxin Penitrem A that is poisonous to humans: therefore, any data that indicates even a moderate inhibitory activity has to be carefully considered.

The absence of the antibacterial activity of digestates can be attributed to intensive proteolysis and to mutual effects of digestive enzymes. Namely, it was shown that intensive proteolysis (>30%) during cheese ripening voided cheese extracts of antibacterial peptides [59]. Additionally, Lopez-Exposito et al. [60,61] showed that the antimicrobial activity produced by the hydrolysis of pure caseins with pepsin digestion was partially or completely lost when the caseins were digested for a period longer than 30 min and 2 h respectively. In addition, the sequential digestion of caseins with pepsin and a mixture of trypsin and chymotrypsin has yielded inactive digests, while the caseins had generated antimicrobial peptides when digested separately with the same enzymes.

## 4. Conclusions

The results of this study showed significant but different antioxidant, ACE-inhibitory and antimicrobial potentials of traditional Serbian white-brined cheeses. The best total antioxidant capacity before digestion was from sheep cheese from Svrljig, whereas the best iron(II) chelating ability was expressed by cow cheese from Sjenica. The observed differences are the result of different contents and compositions of the major contributors such as proteins, peptides and free amino acids. In vitro digestion effected the antioxidant properties of investigated cheeses differently. Digestion dramatically improves the antioxidant capacity of cheeses by 8.42–58.56 times and the reducing power by 17.90–99.30%. Opposite to this, digested cheeses had an unchanged or slightly reduced chelating ability. The major compounds responsible for the antioxidant properties of digested cheeses are low molecular weight peptides and free amino acids. Opposite to the antioxidant properties, in vitro digestion decreased the ACE-inhibitory potential of water-soluble fractions, whereas digestates of water-insoluble fractions are the major source of ACE-inhibitory peptides. These results are of great importance and clearly indicate that besides having other properties, traditional white cheeses may have a significant role in the maintenance of human antioxidant and antihypertensive defense systems. However, further investigation related to the characterisation of peptides responsible for these activities and their stability under in vivo digestion should be conducted.

## Figures and Tables

**Table 1:** The chemical composition and ripening parameters of traditional Serbian white cheeses *.

Parameter	Cow Cheeses			Sheep Cheese		
	Homolje	Sjenica	Zlatar	Homolje	Sjenica	Svrljig
**DM (%)**	59.09 ± 0.05 ^a^	46.81 ± 0.11 ^d^	43.08 ± 5.88 ^d,e^	57.49 ± 0.12 ^b^	51.50 ± 0.01 ^c^	51.82 ± 5.35 ^c,e^
**Fat (%)**	31.25 ± 0.35 ^a^	27.5 ± 0.07 ^c,f^	22.33 ± 4.80 ^f^	30.50 ± 0.05 ^b^	31.50 ± 0.05 ^a^	29.38 ± 3.90 ^a,b,c^
**FDM (%)**	52.89 ± 0.55 ^e,d^	58.75 ± 0.13 ^b^	51.31 ± 4.02 ^e,d^	53.06 ± 0.11 ^d^	61.17 ± 0.01 ^a^	56.56 ± 1.78 ^b,c^
**MNFS (%)**	59.51 ± 0.23 ^e^	73.37 ± 0.15 ^a^	73.09 ± 3.16 ^a,b^	61.17 ± 0,17 ^d^	70.81 ± 0.01 ^b^	68.07 ± 3.84 ^c^
**TN (%)**	3.2338 ± 0.2209 ^a^	2.8223 ± 0.0107 ^c^	2.5022 ± 0.2573 ^d^	2.8976 ± 0.0245 ^b,c^	2.9305 ± 0.0418 ^b^	2.7522 ± 0.0699 ^c^
**TP (%)**	20.63 ± 1.41 ^a^	18.01 ± 0.06 ^c^	16.11 ± 1.78 ^c,d^	18.49 ± 0.16 ^b^	18.70 ± 0.27 ^b^	17.56 ± 0.45 ^c,d^
**WSN (%)**	0.2376 ± 0.011 ^b^	0.3319 ± 0.0072 ^a^	0.3650 ± 0.0725 ^a^	0.2330 ± 0.0136 ^b^	0.3752 ± 0.0092 ^a^	0.3327 ± 0.0646 ^a^
**RI (%)**	7.35 ± 0.29 ^b^	11.76 ± 0.30 ^a^	14.59 ± 2.56 ^a^	8.04 ± 0.46 ^b^	12.80 ± 0.50 ^a^	12.09 ± 2.32 ^a^
**PTA-SN (%)**	0.0752 ± 0.0002 ^c^	0.0511 ± 0.0013 ^e^	0.0217 ± 0.0065 ^g^	0.0359 ± 0.0002 ^f^	0.1050 ± 0.0047 ^a^	0.0761 ± 0.0070 ^c^
**TCA-SN (%)**	0.1756 ± 0.0046 ^f^	0.2597 ± 0.0046 ^c^	0.1981 ± 0.0114 ^e^	0.2315 ± 0.0011 ^d^	0.3246 ± 0.034 ^a^	0.2351 ± 0.0332 ^d^
**NaCl (%)**	5.43 ± 0.01 ^c^	3.69 ± 0.03 ^f^	3.48 ± 0.27 ^f^	6.49 ± 0.31 ^a^	4.13 ± 0.01 ^e^	5.95 ± 0.01 ^b^
**pH**	4.71 ± 0.03 ^a^	4.51 ± 0.09 ^b^	4.69 ± 0.19 ^a^	5.18 ± 0.24 ^a^	4.29 ± 0.11 ^b^	4.88 ± 0.29 ^a^

* Data within the same row marked with different lowercase letters are significantly different at *p* ˂ 0.05; DM, dry matter; FDM, fat in dry matter; MNFS, moisture in nonfat solid; TN, total nitrogen content; TP, total protein content; WSN, water soluble nitrogen; RI, ripening index; PTA-SN, content of nitrogen soluble in phosphotungistic acid; TCA-SN, content of nitrogen soluble in trichloracetic acid.

**Table 2:** The antioxidant properties of traditional white-brined cheeses and their digestates *.

Cheese	TEAC mmol Trolox (Eq/kg)		Reducing Power A_700_		Chelating Properties IC_50_ (mg/mL)	
	B.D.	A.D.	B.D.	A.D.	B.D.	A.D.
Sjenica						
sheep	17.13 ± 1.36 ^b,B^	400.1 ± 9.44 ^a,A^	0.143 ^e,B^	0.285 ^c,A^	42.34 ± 1.3 ^a,A^	39.34 ± 0.95 ^b,A^
cow	16.54 ± 0.81 ^b,B^	209.53 ± 5.42 ^e,c,A^	0.296 ^a,B^	0.349 ^a,A^	36.15 ± 0.89 ^b,B^	40.31 ± 1.10 ^b,A^
Homolje						
sheep	5.82 ± 2.02 ^g,B^	348.80 ± 15.17 ^b,A^	0.150 ^d,e,B^	0.269 ^d,A^	31.34 ± 0.45 ^c,B^	38.59 ± 1.42 ^b,A^
cow	8.81 ± 1.96 ^e,f,B^	262.3 ± 9.01 ^d,A^	0.156 ^c,d,B^	0.194 ^g,A^	15.19 ± 0.67 ^e,B^	29.87 ± 1.21 ^c,A^
Svrljig						
Sheep	36.43 ± 0.15 ^a,B^	306.94 ± 13.16 ^c,A^	0.136 ^f,B^	0.255 ^e,A^	29.71 ± 1.2 ^c,A^	27.53 ± 1.15 ^c,A^
Zlatar						
Cow	10.28 ± 0.27 ^d,f,B^	250.40 ± 4.92 ^d,A^	0.176 ^b,B^	0.285 ^b,A^	34.22 ± 1.20 ^b,B^	42.80 ± 0.63 ^a,A^

TEAC, Trolox equivalent antioxidant capacity; IC_50_, protein concentration needed to chelate half of the added Fe(II) ions; B.D., before digestion; A.D., after digestion; * data within the same column marked with different lowercase letters are significantly different at *p* ˂ 0.05; data within the same row and the same parameter marked with different uppercase letters are significantly different at *p* ˂ 0.05.

**Table 3:** The angiotensin-converting enyzme-inhibitory (ACE-inhibitory) activity of protein fractions of traditional Serbian white cheeses *.

Protein Fraction		ACE-Inhibitor Activity * IC_50_ (mg/mL)					
		Sheep Cheeses			Cow Cheeses		
		Svrljig	Homolje	Sjenica	Homolje	Sjenica	Zlatar
WSF	b.d.	3.87 ± 0.05 ^g^	4.50 ± 0.24 ^e^	4.61 ± 0.04 ^e^	2.95 ± 0.01 ^i^	3.79 ± 0.06 ^g^	2.26 ± 0.02 ^j^
	a.d.	7.09 ± 0.11 ^b^	8.96 ± 0.014 ^a^	4.71 ± 0.01 ^e^	4.73 ± 0.08 ^e^	4.26 ± 0.02 ^f^	5.63 ± 0.11 ^d^
WINF	a.d.	6.39 ± 0.21 ^c^	5.84 ± 0.07 ^d^	3.28 ± 0.12 ^h^	4.20 ± 0.23 ^e^	3.17 ± 0.07 ^h^	1.31 ± 0.07 ^k^
			ACE inhibition (%) **				
WSF	b.d.	64.23 ± 0.10 ^e^	55.10 ± 0.21 ^g^	54.23 ± 0.54 ^g^	69.20 ± 0.42 ^d^	64.96 ± 0.35 ^e^	75.26 ± 0.41 ^b^
	a.d.	35.27 ± 0.05 ^m^	32.40 ± 0.35 ^k^	53.53 ± 0.67 ^g,h^	52.85 ± 0.36 ^h^	57.68 ± 0.24 ^f^	46.40 ± 0.18 ^i^
WINF	a.d.	39.45 ± 0.15 ^l^	44.80 ± 0.22 ^j^	71.22 ± 0.11 ^c^	54.52 ± 0.54 ^g^	71.86 ± 0.52 ^c^	84.23 ± 0.32 ^a^

The average values ± the standard deviation of the IC_50_ values (expressed as mg protein fraction per mL test solution that inhibited the ACE activity by 50% in the in vitro assay). * Data with different lowercase letters are significantly (*p* ˂ 0.05) different: ** the inhibition percent of peptide solutions (5 mg/mL). WSF: The water-soluble protein fractions; WINF: water-insoluble protein fractions.

**Table 4:** The antifungal activity of cheese digestates and their fractions (mg/mL).

		Sjenica Cow Cheese			Svrljig Sheep Cheese			Homolje Cow Cheese		
		WINF	WSF	Cheese	WINF	WSF	Cheese	WINF	WSF	Cheese
mg/mL										
Micromycetes										
*Aspergillus fumigatus*	MIC	4.80	n.d.	4.40	4.00	4.00	4.00	4.16	4.64	4.00
	MFC	n.d.	n.d.	n.d.	n.d.	n.d.	n.d.	n.d.	n.d.	n.d.
*Aspergillus versicolor*	MIC	4.80	5.00	4.40	2.00	4.00	2.00	2.08	4.64	4.00
	MFC	n.d.	n.d.	n.d.	4.00	n.d.	4.00	4.16	n.d.	n.d.
*Aspergillus ochraceus*	MIC	4.80	5.00	4.40	4.00	4.00	4.00	4.16	4.64	4.00
	MFC	n.d.	n.d.	n.d.	n.d.	n.d.	n.d.	n.d.	n.d.	n.d.
*Aspergillus niger*	MIC	n.d.	n.d.	n.d.	n.d.	n.d.	4.00	n.d.	4.64	n.d.
	MFC	n.d.	n.d.	n.d.	n.d.	n.d.	n.d.	n.d.	n.d.	n.d.
*Trichoderma viride*	MIC	4.80	n.d.	n.d.	n.d.	n.d.	4.00	4.16	n.d.	n.d.
	MFC	n.d.	n.d.	n.d.	n.d.	n.d.	n.d.	n.d.	n.d.	n.d.
*Penicillium funiculosum*	MIC	n.d.	n.d.	n.d.	n.d.	n.d.	n.d.	4.16	2.32	n.d.
	MFC	n.d.	n.d.	n.d.	n.d.	n.d.	n.d.	n.d.	4.64	n.d.
*Penicillium ochrochloron*	MIC	n.d.	n.d.	n.d.	n.d.	n.d.	n.d.	n.d.	2.32	n.d.
	MFC	n.d.	n.d.	n.d.	n.d.	n.d.	n.d.	n.d.	4.64	n.d.

MIC, minimum inhibitory concentration; MFC, minimum fungicidal concentration. n.d.—not detected
